# Subcellular mechano-regulation of cell migration in confined extracellular microenvironment

**DOI:** 10.1063/5.0185377

**Published:** 2023-12-29

**Authors:** Daesan Kim, Dong-Hwee Kim

**Affiliations:** 1 KU-KIST Graduate School of Converging Science and Technology, Korea University, Seoul 02841, Republic of Korea; 2 Department of Integrative Energy Engineering, College of Engineering, Korea University, Seoul 02841, Republic of Korea; 3 Biomedical Research Center, Korea Institute of Science and Technology, Seoul 02792, Republic of Korea

## Abstract

Cell migration is a highly coordinated cellular event that determines diverse physiological and pathological processes in which the continuous interaction of a migrating cell with neighboring cells or the extracellular matrix is regulated by the physical setting of the extracellular microenvironment. In confined spaces, cell migration occurs differently compared to unconfined open spaces owing to the additional forces that limit cell motility, which create a driving bias for cells to invade the confined space, resulting in a distinct cell motility process compared to what is expected in open spaces. Moreover, cells in confined environments can be subjected to elevated mechanical compression, which causes physical stimuli and activates the damage repair cycle in the cell, including the DNA in the nucleus. Although cells have a self-restoring system to repair damage from the cell membrane to the genetic components of the nucleus, this process may result in genetic and/or epigenetic alterations that can increase the risk of the progression of diverse diseases, such as cancer and immune disorders. Furthermore, there has been a shift in the paradigm of bioengineering from the development of new biomaterials to controlling biophysical cues and fine-tuning cell behaviors to cure damaged/diseased tissues. The external physical cues perceived by cells are transduced along the mechanosensitive machinery, which is further channeled into the nucleus through subcellular molecular linkages of the nucleoskeleton and cytoskeleton or the biochemical translocation of transcription factors. Thus, external cues can directly or indirectly regulate genetic transcriptional processes and nuclear mechanics, ultimately determining cell fate. In this review, we discuss the importance of the biophysical cues, response mechanisms, and mechanical models of cell migration in confined environments. We also discuss the effect of force-dependent deformation of subcellular components, specifically focusing on subnuclear organelles, such as nuclear membranes and chromosomal organization. This review will provide a biophysical perspective on cancer progression and metastasis as well as abnormal cellular proliferation.

## I. INTRODUCTION

Currently, several studies aim to comprehensively elucidate the physiological characteristics of cells. Notably, mechanobiology has been located at the forefront of emerging research areas over the years, presenting noteworthy advancements in addressing unresolved inquiries.^1–3^ Mechanobiology investigates the changes in the intrinsic properties of cells under external forces, providing a new perspective by incorporating physical methodologies into biology and its application fields.

To explore biological characteristics, researchers have focused on cell movement, which showcases fundamental features and helps elucidate intuitive changes.^4–10^ In particular, studying cell migration is key to understanding cell dynamics and a comprehensive demonstration of physiological responses. While basic cell movement is influenced by intrinsic cell properties, in many cases, it is also significantly affected by various external factors largely stemming from interactions between cell and extracellular microenvironment. To identify such causality, considerable efforts have been made, and mechanistic models that can simulate cell movement have been researched, enabling the analysis of both intrinsic cell movement and how cells respond to and change under external forces.^11–15^ This opens up the possibility of anticipating cellular changes and designing experiments in advance.

Specifically, the differences in the phenomena were compared by distinguishing between mechanical deformation and protein degradation, which can withstand external forces. Permanent deformation includes gene expression and protein changes in cells caused by cell nucleus rupture, which often occurs in the actual internal environment of living organisms, necessitating further research in the field. To investigate these phenomena, it is necessary to examine cell movement in a confined physical setting. By studying the changes exhibited by cells in such a confined microenvironment, we can not only research the impact of the surrounding environment on cells but also directly examine the mechanisms of cell movement, such as the relationship between the physical factors acting on the cell nucleus and gene expression, and the intrinsic characteristics of cells provide excellent examples.

In this review, we examine the effects of physical stress on cells and, specifically, the responses and changes at the gene level, as well as aspects related to cell fate. Finally, we explored current topics of great interest and identified future research directions and unresolved questions.

## II. CELL MIGRATION PRINCIPLES IN COMPLEX ENVIRONMENTS

Cell migration plays a pivotal role in various physiological and pathological processes including embryonic development, wound healing, and cancer metastasis. This phenomenon can be examined from two distinct perspectives: single cell migration, which focuses on the characteristics of individual cellular movements, and collective cell migration, which involves the coordinated movement of multiple cells. The investigation of collectively migrating cells has addressed the limitations in traditional single cell studies and has endeavored to explore cellular behavior within realistic environmental contexts. While collective cell migration is physiologically relevant,^16^ we chose to focus on single cell migration, which enables us to elucidate the effects of different physical cues at the resolution of the single cell level in the absence of other adherent cells.

### A. Cell migration mode in dimensional views

**FIG. 1.**
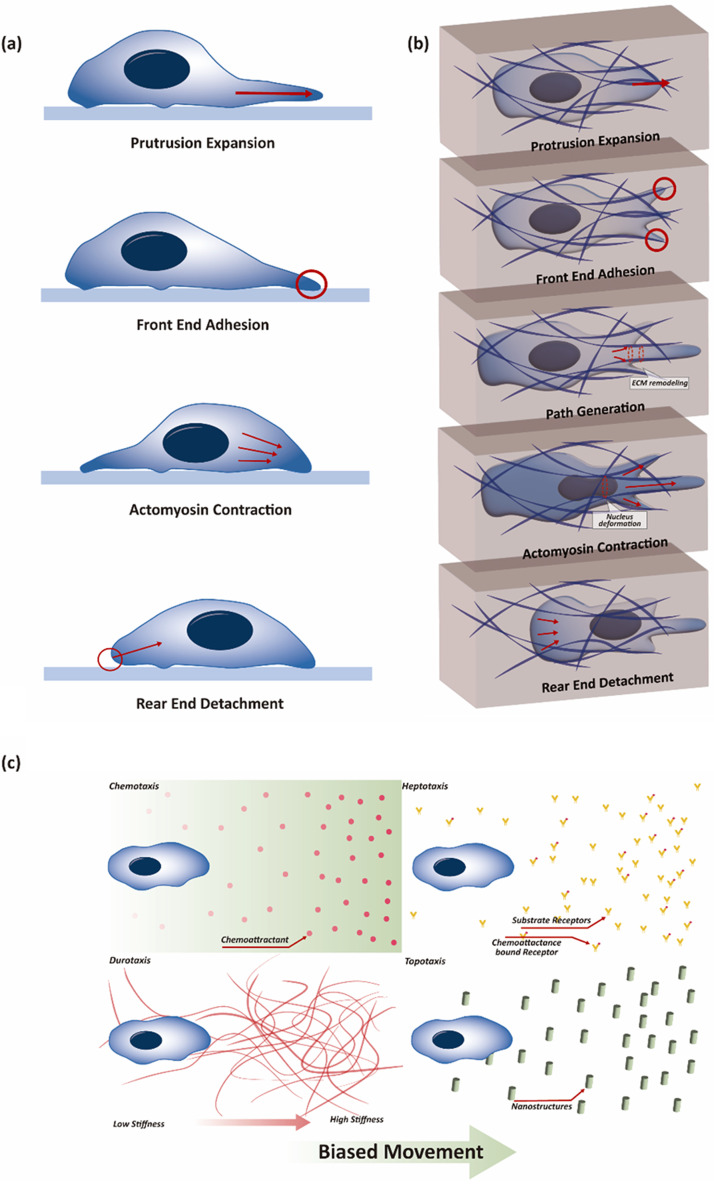
Cell migration according to different prospectives. (a) and (b) Cell migration stages in 2D and 3D environments. Cell migration varies between 2D (a) and 3D (b) environments, with the major contrast being the presence of obstacles in 3D. In 3D, cells need to actively generate a migration path to facilitate their movement. Aside from this path formation, the fundamental mechanism of cell migration remains largely consistent between the two environments. (c) Cell migration guided by various cues. Cell movement is influenced by diverse cues that guide directed migration. Among these, four well-established cell behaviors are noteworthy. Chemotaxis, the most fundamental signaling system, is triggered by chemical signals. Haptotaxis directs cell movement based on the chemical signals present in the adherence substrate or the gradient of the adherence site. In contrast, the other two mechanisms involve mechanical signals. Durotaxis dictates the direction of cell movement based on substrate strain, while topotaxis relies on the topographic gradient to govern cell movement. The regulation of cell migration is a complex decision-making process, influenced by a combination of various criteria rather than being solely determined by any one factor.

Cell migration varies depending on environmental condition and cell type. However, in a simple scenario, the migration process can be universally classified into two modes. One involves the production or support of mechanical forces such as protrusion and retraction of the cell membrane edge. The other involves the construction or disruption of protein bonding between binding proteins and substrates.^17^ These migratory modes vary only slightly in two-dimensional (2D) and three-dimensional (3D) environments.^18,19^ In 2D conditions, cells move under the assumption of an infinite spatial condition, whereas in 3D settings, they encounter numerous obstacles. Therefore, in 3D environments, the cells must navigate around obstacles or create alternative pathways. Simplified explanations can be provided as follows: in 2D, migration is depicted by four stages, whereas in 3D, it involves five stages [Figs. 1(a) and 1(b)]: (1) Protrusion phase: Cell migration is initiated in the protrusion phase, where cells polarize and extend protrusions compromising actin filaments, thereby propelling the cell forward by pushing against the plasma membrane. (2) Interaction with the extracellular matrix (ECM) phase: In this stage, as cells advance, they interact with the surrounding ECM to provide both physical support and essential guidance cues. (3) Migration track path formation phase (only in 3D): Migration in 3D environments includes the formation and adaptation of the migration track path. This occurs through proteolytic degradation and realignment of the ECM, which enables cells to create passages. (4) Actomyosin contraction phase: In the subsequent actomyosin contraction phase, cells exert contractile forces primarily at their trailing edges, thereby facilitating forward movement across diverse environments. (5) Release of the rear adhesion sites phase: Finally, cells progress to the release of the rear adhesion sites. During this stage, the cells strategically release the rear adhesion sites, allowing for efficient sliding along the substrate.

### B. Cell migration with directionality by environmental cues

Cell migration has directionality, in which cells move in their desired direction for various purpose. This directionality largely depends on the specific physical cues, such as chemical or physical stimuli.^20,21^ To understand single cell migration, it is essential to explore directional migration driven by diverse cues [Fig. 1(c)]. While directional migration has been a subject of research for over a century and many aspects still remain unknown, accumulating evidence reveal that directional migration is mediated by various inducible signals and pathways associated with specific receptors.^21,22^ Chemotaxis, for instance, involves cell movement directed by a chemical gradient cue, which is well-known for its classical signaling pathway mediated by G protein-coupled receptors (GPCRs).^23,24^ Chemotaxis operates based on the chemical gradient cue within a solution, whereas haptotaxis relies on the cell binding substrate. Haptotaxis involves sensing the gradient of cell adhesion sites or chemical cues on the surface of these adhesion sites.^21,25,26^ As immobile chemical cues on the surface of the cell binding substrate or ECM play a crucial role in signal transmission, the integrin-mediated signaling pathway is the primary signaling system.

Although certain aspects of the signaling pathways and sensing components involved in directional migration are reasonably well understood, many characteristics within this realm remain to be elucidated. In contrast to chemotaxis and haptotaxis, where chemical cues predominantly guide cell movement, the characteristics of mechanical cue-based migration, such as durotaxis and topotaxis, are less understood. Durotaxis relies on cellular sensing of mechanical characteristics in extracellular micro-environmental settings, where the substrate stiffness determines the direction of cell migration.^27^ On stiff substrates, cells may exhibit distinct elongated characteristics, as observed in fibroblasts. Researchers have shown considerable interest in the use of mechanical factors, such as in substrate engineering, to control cell migration.

Similarly, topotaxis relies on direct mechanical cues, with cells perceiving topographic gradients on substrates.^28^ While research in this phenomena is still in its early stages, the interest in utilizing mechanical cue-based migration, alongside surface engineering, has grown significantly.

Although chemical cue-driven migrations, such as chemotaxis and haptotaxis, have well-defined mechanisms and signaling pathways, migrations based on mechanical cues, such as durotaxis and topotaxis, are relatively less understood. However, recent efforts in mechanobiology have generated substantial interest and hold promise for numerous research and practical applications.

### C. Cell migration mode by mechanical views

Single cell migration can be broadly categorized into two main modes based on its dynamic features: mesenchymal migration and amoeboid migration. A notable distinction between these modes lies in the direction of cell movement and the positioning of the cell nucleus. In mesenchymal migration, the nucleus typically assumes a posterior position within the cell, facilitating passive propulsion through actomyosin contractility, reminiscent of slingshot motion. In contrast, amoeboid migration involves the nucleus situated toward the anterior of the cell, facilitating active forward movement along the migration axis.

Furthermore, there was a notable difference in the migration speeds between these modes. Amoeboid migration was approximately ten times faster than mesenchymal migration.^29,30^ This phenomenon is affected by cell adhesion properties. During amoeboid migration, a rapid adhesion/detachment cycle is formed due to weak interactions with the substrates, ultimately resulting in faster migration.^31^ Additionally, strong polarization of actin appears to enhance these dynamics.^32^ Conversely, during mesenchymal migration, strong integrin-mediated adhesion creates resistance to movement. Because protrusions occur in multiple directions, resulting in energy dissipation owing to their competition, migration speed can be reduced, whereas cells under lateral confinement exhibit the enhanced motility.^33^

Notably, these behaviors are not fixed within a single cell but vary depending on the environment. Under conditions of strong substrate adhesion, cells typically exhibit mesenchymal migration. However, when faced with environments where adhesion is weakened, such as in confined environments,^34^ cells form migration pathways by degrading ECM using matrix metalloproteinases (MMPs).^4,35^ In cases where such actions are difficult, cells may transition from mesenchymal migration to amoeboid migration. Therefore, when considering scenarios such as cancer cell metastasis, where migration occurs in micro-confined environments, the dynamic transitions between migration modes should be considered.

## III. MECHANISTIC ANALYSIS OF CELL DYNAMICS

Mechanical modeling has become an essential tool for studying the intricate mechanics of cells and their roles in various biological processes. Here, we provide a comprehensive overview of the mechanical models employed to investigate cellular behavior, focusing on the principles, methodologies, and applications of these models. In light of rapid advancements in simulation technology, the field of cell modeling has undergone significant changes. However, a foundational understanding is crucial to effectively harness the potential of these evolving technologies. Our exploration extended to the fundamental mechanical properties of cells, including elasticity, viscosity, and adhesion, and elucidated the integration of these properties into mechanical models. Various modeling approaches, such as continuum models, microstructural network models, and particle-based methods, have been explored along with their strengths and limitations. In addition, we examined the application of mechanical models to understand nuclear deformation, cytoskeletal dynamics, and mechanotransduction. The mechanical modeling of cells can be broadly categorized into three main approaches.

### A. Continuum approach models

The first approach assumes the entire cell is a continuous structure, in which the material properties of each part are connected continuously. Among these modeling methods, the most widely used is the linear viscoelastic model.^36–38^ This model separates the viscoelastic properties of the cell cytoplasm and nucleus and defines the characteristics of each part individually. Each part is then analyzed as a single component using a linear combination. In the past, attempts have been made to apply different physical properties; however, current experimental results have established that the physical properties of cells exhibit viscoelastic behaviors.^39^ Few attempts have been made to deviate significantly from these assumptions. However, there were variations in the detailed approaches. While some models use the simplest viscoelastic model, the Kelvin–Voigt model, to represent the physical characteristics of each part, others use the standard linear solid model (or the Zener model) for a more detailed representation.^40,41^ The latter is generally preferred for obtaining more accurate results; however, it exhibits mathematical complexities under certain conditions. In the case of a significant error in the analysis using the simplified model above, the characteristics of the cell were scrutinized using the Burgers model.^15^ This model is employed to depict the impact of the presence or absence of components, such as lamin inside the cell nucleus, on nuclear deformation. The creep compliance *J(t)* (strain/stress) is expressed by the following equation using the Burgers model:^15^

Jt=1Km+1Kkv1−etτ+tμm,

where elasticities of the springs and viscosities of dampers are depicted by $K_{m},\;K_{kv}$ and $\mu_{m},\;\mu_{kv}$, respectively, and calculated the physical parameter is defined as $\tau =\frac{\mu_{kv}}{K_{kv}}$, where the deformation dynamics of cell was well matched with experimental results via *J(t)*.

In mechanobiological perspectives, the motor-clutch model has been employed to study the transmission of force to cells.^42–44^ This model assesses the force transmitted to cells based on the mechanical characteristics of the surrounding environment by utilizing a clutch model that physically connects F-actin and extracellular substances. Its application extends to the analysis of cell migration, enabling the prediction of cell movement in 2D space through a continuum model.^43^ Specifically, this model has been instrumental in investigating cell movement within a one-dimensional environment, such as a confined environment. The unbinding point of the *i*th clutch was calculated using single exponential of the rate *k_off,i_*,^45^

$$k_{\text{off},i}=k_{\text{off}}\cdot e^{\frac{F_{\text{clutch},\;i}}{F_{\text{bond}}}}.$$

Here, the unloaded unbinding rate is *k_off_*, the characteristic bond force is *F_bond_*, and the force on the *i*th clutch is *F_clutch,i_*, which predicts the detachment of the adhesion site.

Nevertheless, certain studies have concentrated on modeling with an emphasis on elasticity. This modeling approach is frequently employed to observe nuclear deformation during cell migration within a confined space. Utilizing a continuum elastic model to simulate nuclear deformation within a confined channel, the analysis sought to investigate the factors influencing deformation in a two-dimensional context and to quantify the force exerted on the surface of the cell nucleus.^46^

Consequently, an analysis based on existing research findings indicated that Arp2/3 plays a role in mediating actin polymerization to facilitate the passage of cell nuclei through physically confined spaces.^47^ This model predicts the distribution of force alignment with the direction of motion. The forces around the nucleus act perpendicularly to the direction of motion, indicating the necessity for lateral forces to compress the nucleus and direct it into a confined channel. The perpendicular force surrounding the nucleus is linked to Arp2/3-mediated perinuclear actin network assembly. Furthermore, the anticipated model of actin polymerization force passing through the constrained channel is similarly utilized to elucidate the alterations in the cytoplasm.^48^

### B. Microstructural approach models

The second approach does not consider the cell components as a continuous structure but instead views the various filaments within the cell to form a network structure. This modeling approach allows for a more intuitive and direct calculation of responses to physical stimuli transmitted through filaments.^49^ However, compared to the previously mentioned models, it introduces increased complexity. As each filament (actin, intermediate filaments, and microtubules) has distinct physical properties, modeling based on single-filament characteristics can lead to issues. Additionally, it may be challenging to consider critical perturbations due to thermal energy at the microscale level. Nonetheless, there are models that address these drawbacks, e.g., the coarse-grained model, which offers improved solutions, and various modeling and analysis methods are being developed.^50,51^ Because these models are typically based on thermodynamics, a strain energy function can be simplified at 1D (W_1D_) as follows:^52^

$$W_{1D}=W_{b}+W_{s},$$

$$W_{b}=\frac{\mu_{b}}{4}{\left(\lambda_{f}-1\right)}^{2},\;W_{s}=\frac{\mu_{s}}{2}\int_{1}^{\lambda_{s}}f_{s}\left({\lambda^{\prime }}_{s}\right)d{\lambda \prime }_{s},$$

where W_b_ and W_s_ are the strain energies generated by filament bending and stretching, respectively. $\mu_{b},\;\mu_{s}$ and $\lambda_{b},\;\lambda_{s}$ are the stiffness parameters and magnitudes of bending and stretching of individual filament, respectively.

The cytoskeletons of the sophisticated cells were represented through the interconnection of the elastic cable network model.^53^ The kinetics of the cells were evaluated through methodologies such as cell poking, magnetic twisting cytometry, and magnetic bead micrography, allowing for comparative analysis.

However, treating an entire cell as a singular continuum body has limitations across several dimensions. Specifically, the single-continuum-body model faces challenges when attempting to capture anisotropic motion, which involves movement in a specific direction or protrusions occurring in various directions. To address this limitation, cell dynamics were analyzed under the assumption that cell protrusion, induced by interactions with the ECM constitutes a unidirectional mechanical system. This model facilitates the successful study of cell dynamics by incorporating chemosensing responses and ECM deformation.^54^

In contrast to earlier discussions, the crucial microstructure in the biological realm extends beyond the confines of the cell interior. Notably, the ECM, which directly engages cells, is a critical mediator determining cellular mechanics.^55,56^ However, given its distinct characteristics, concurrently considering both poses numerous challenges. Some studies underscore the significance of modeling that directs attention toward the microenvironment surrounding the cell, rather than exclusively concentrating on internal cellular dynamics;^57^ in these studies, cells are simplified and modeled as elastic spheres, while the microstructure of the ECM network is meticulously simulated to replicate fiber-mediated force transfer.

This approach successfully demonstrated the implementation of durotaxis characteristics in MCF-10A cells through simulation. As the cell undergoes movement, it pulls the ECM fiber, which cause the ECM deformation. Therefore, the stiffer fiber network causes less deformation than the soft fiber network. These unique characteristics contribute to an increased propensity for locomotion in the direction of the stripper fiber,^57^

$$\delta x=\text{max}\left\{\delta l-\left(d-d^{\prime }\right),0\right\}\cdot d_{0}^{\prime },$$

where δx, δl are the contraction of the filament by cell and intrinsic property, respectively. *d* and d′ distance between the cell center and the adhesion node before and after ECM deformation, respectively. $d_{0}^{\prime }$ is the direction vector of the ECM deformation. The accumulation of these effects leads to the localized migration to the overall biased migration. This phenomenon is examined as a key factor explaining the observed tendency of cells to move in alignment with stiffness gradients in experimental settings.

### C. Particle-based dynamics models

Particle-based dynamics model assumes that each part of the cell is composed of point or mass particles.^58,59^ This approach calculates the forces and changes acting on each particle according to Newton's second law of motion. Depending on the modeling objective, it allows for the consideration of the chemical bonding strength between particles and the participation of various substances in different parts. The results were derived from the Navier–Stokes equation without considering the mesh structures, which provides the benefit of directly understanding the interpretation results and physical implications of the parameters. However, the advantage of a straightforward physical interpretation comes with the challenge of solving the Navier–Stokes equation during the analysis process. Therefore, as the scale of modeling increases with the number of particles, the analysis becomes more challenging.

A study of erythrocyte dynamics in tube flow, observing the behavior of individual cells, was conducted using dissipative particle dynamics (DPD).^60,61^ This study employed DPD to simulate particle dynamics, assuming compliance with Newton's second law for all particles.

$$m_{i}\frac{d^{2}r_{i}}{dt^{2}}=\sum_{j\neq i,\;j\in P_{A}}f_{ij}^{\mathrm{DPD}},\;i\in P_{F}\cup P_{I},$$

$$m_{i}\frac{d^{2}r_{i}}{dt^{2}}=\sum_{j\neq i,\;j\in P_{A}}f_{ij}^{\mathrm{DPD}}+f_{i}^{\mathrm{Mem}}+f_{i}^{\mathrm{Int}},\;i\in P_{M},$$

where *m_i_* and *r_i_* are the mass and location of *i*th particle, respectively. The particles were collected in the set $P_{A}$, where $P_{A}=P_{M}\cup P_{F}\cup P_{I}\cup P_{W}$. The subscripts M, F, I, and W below *P* indicate membrane, fluid, cytoplasm, and wall particles, respectively. This particle-based analysis demonstrated a notable advantage in discerning the distinct characteristics of individual cells.

For instance, this model successfully investigated the aggregation or deformation interactions between both healthy and diseased red blood cells (RBCs).^60^ The deformation and aggregation of cells significantly influence the initial shape and mechanical properties of cells. When examining diseased RBCs, such as those infected with malaria, distinct variations in shape and properties are observed. In the study of mixed healthy and diseased RBCs in tube flow, the results differ from those obtained by studying only healthy RBC groups. This disparity is attributed to the variations in intercellular interaction between healthy and diseased red blood cells.

## IV. UNIQUE LOCOMOTIVE CHARACTERISTICS OF CELL DURING MIGRATION UNDER CONFINEMENT ENVIRONMENT

Cell migration in a confined environment is more complicated than that in a unconfined open space because it is tightly regulated by intricate interactions between the mechanical properties of the cells and their surrounding microenvironment. It involves unique forces and motions that are particularly relevant for navigating through tight spaces or constrictions. While these forces and motions can also play a role in cell migration under non-confined conditions, they become more pronounced and critical under confined conditions.

### A. Hydraulic effect on cell migration under confinement environment

**FIG. 2.**
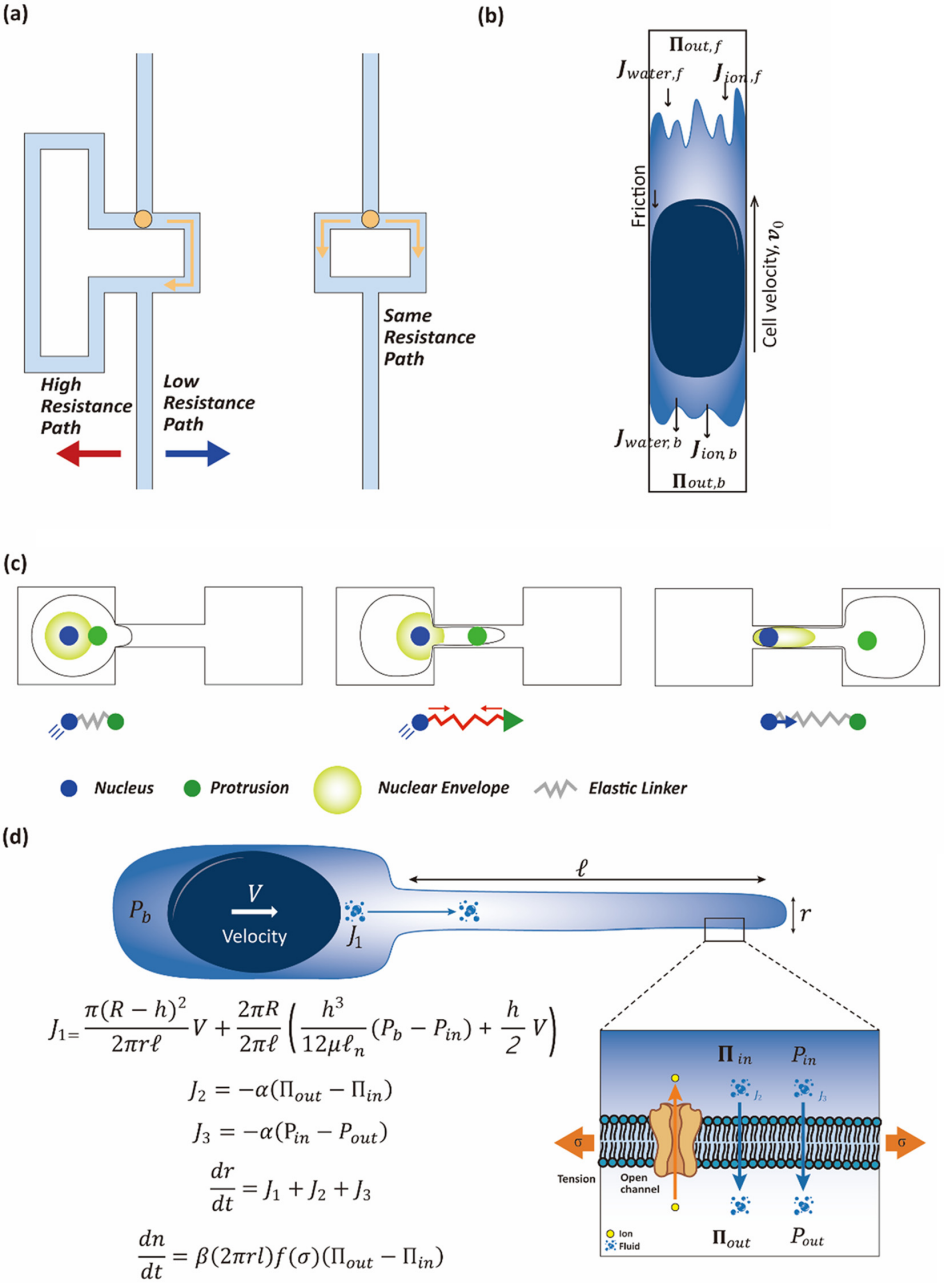
Distinct cell migration locomotion in micro-confinement environments. Cell migration in controlled environments involves specialized mechanisms for generating driving forces to navigate obstacles, exhibiting movements that differ from those in obstacle-free spaces. (a) For cells conditioned within multiple confined pathways, hydraulic resistance induces movement in a downward direction, facilitating efficient cell migration (barotaxis). (b) Cells confined to a single pathway leverage differences in osmotic pressure at the front, back, and inside the cell to generate an additional driving force, thereby generating movement. (c) and (d) A more mechanical perspective on cell movement in defined conditions reveals two distinctive features. One method involves explaining cell movement through a slingshot motion (c), emphasizing the elastic aspect of the cell body connected to the cell nucleus. Another explanation involves the utilization of the nuclear piston model (d), depicting the cell nucleus as a piston that propels the front end of the cell.

In a confined environment, several physical cues influence cell migration, resulting in distinct movements and driving forces. In order to analyze constrained cell migration with various water pressures, the unique characteristics of constrained cells under asymmetric geometry were observed.^6^ In this study, the role of hydraulic resistance as a physical input to cellular directionality during cell migration in confined environments was investigated, where the hydraulic resistance indicates that the extracellular resistance to water flow.^62^ Cells in microchannels exhibit biased migration toward the path of lesser hydraulic resistance, termed barotaxis, when presented with asymmetric hydraulic environments [Fig. 2(a)]. These findings suggest that cells integrate chemotactic and barotactic cues to navigate intricate environments, thereby reducing the likelihood of local entrapment.

In studies focused on understanding the impact of hydraulic resistance on cancer metastasis, diverse geometries were employed using microfluidic devices to establish a migration path featuring distinct hydraulic resistances.^63^ This setup was developed to closely examine the directional orientation of the cells. Upon comparing the movement patterns along each route, using both non-metastatic cells (MCF-7) and metastatic cells (MDA-MB-231), the findings, in line with earlier studies, indicated a clear preference for pathways characterized by low hydraulic resistance.^6^ This outcome underscores the notion that the more significant the difference in resistance, the more pronounced is the shift in movement tendencies. Specifically, research indicates that within a confined environment, cells do not gravitate toward the lower hydraulic resistance between two paths; instead, they actively select from multiple available paths.^64^ Both breast cancer cell and fibrosarcoma cells (MDA-MB-231 and HT1080) have demonstrated a tendency to move in the direction of the lowest resistance in pathways with varying hydraulic resistances, such as those configured in a cross shape. This experiment highlights that transient receptor potential cation channel subfamily M member 7 (TRPM7), which is a mechanosensitive Ca^2+^ channel and plays a crucial role in intracellular Ca^2+^ homeostasis, serves as a crucial mechanosensing component, and the hydraulic resistance-mediated TRPM7 activation triggers calcium influx. Consequently, it supports a thicker cortical actomyosin meshwork containing the actomyosin motor, guiding the cell toward low resistance channels.^64,65^

The highly sensitive response of TRPM7 to mechanical deformation of the membrane provides a plausible explanation for the preferential involvement of TRPM7 in cell responses triggered by hydraulic pressure, compared to other mechanosensitive ion channels. The pressure sensitivity of TRPM7 is 500 Pa,^66,67^ which is 10-fold lower than other mechanosensitive channels [Piezo1/2 and transient receptor potential cation channel subfamily V member 4 (TRPV4)], ranging from 4000 to 5000 Pa.^68–70^

Hydraulic resistance not only influences the direction of cell movement but also dictates the speed of cell migration within confined spaces.^71^ The detection of alterations in media viscosity and hydraulic resistance, which is proportional to viscosity, facilitated by calcium ion-dependent mechanisms, revealed consequential impacts on the dynamics and speed of cell migration. Notably, this phenomenon is similar to the enhanced movement observed in response to osmotic pressure,^72^ which will be discussed later.

The osmotic engine model is a theoretical concept used to explain cell migration under confined conditions [Fig. 2(b)].^72^ It proposes that cells can utilize osmotic forces to generate water flow across the membrane, which acts as a driving force for cell movement in confined spaces.^73,74^ In confined environments, cells may experience changes in solute concentrations, leading to an osmotic imbalance. These osmotic imbalances result in the influx or efflux of water into the cell, causing it to swell or shrink. As a result, this water flow generates a driving force that aids cell migration.

The osmotic engine model suggests that the imbalance of net flow of water at the cell leading and trailing edge generated osmotic shocks which affect cell motility, facilitating movement through narrow passages or constrictions.^72^ This mechanism provides an alternative means of cell migration without the need for direct physical interactions with the surrounding substrate. Moreover, the osmotic engine model may complement other driving forces, such as actin-based protrusions.^75,76^ The osmotic engine model relies on the balance between hydrostatic pressure and osmotic pressure across the semipermeable cell membrane. In addition to allowing direct permeability to water molecules, cell membranes have various active channels that regulate ion flux and solute. This enables cells to actively adjust osmotic pressure, thus governing water flux at both the front and back of the cells. This highlights the concept that cells can generate driving forces through the control of ion channels. Notably, among these channels, Na^+^/H^+^ exchanger 1 (NHE1) and leucine-rich repeat-containing protein 8a (LRRC8A or SWELL1) play a predominant role in confined channels.^72,77^ NHE1 and SWELL1 have distinct polarization patterns and functions in regulatory volume increase (RVI) and regulatory volume decrease (RVD). NHE1 is involved in RVI and is enriched at the cell leading edge, facilitating an increase in cell volume. On the other hand, SWELL1, positioned at the cell trailing edge, mediates local RVD through the outflow of chloride ions and water, leading to rear membrane shrinkage and decreased cell volume relative to its equilibrium state.

The process involves a feedback loop where, upon exceeding the equilibrium state due to RVI, SWELL1 is reactivated, initiating a coordinated RVD/RVI cycle. This dynamic regulation of water and ion fluxes allows cells to maintain their volume and migrate efficiently in confined spaces. The polarization of SWELL1 at the cell trailing edge plays a key role in local RVD, while the enrichment of NHE1 expression at the cell leading edge supports RVI, collectively contributing to the orchestrated cell migration in response to changing volume conditions. By considering osmotic forces as contributing factors to confined cell migration, the model offers valuable insights into the complex interplay between mechanical cues and physical constraints that influence cell behavior in restrictive environments.

For a more compelling demonstration of motion powered by osmotic engines, the movement of giant liposomes, which have not internal driving force, was examined within microfluidic channels.^78^ The creation of osmotic pressure inside the microchannels through a salt concentration gradient induced momentum for the liposomes' movement. In line with previous studies, this experiment validated that liposomes exhibit the anticipated movement in response to osmotic pressure.

### B. Mechanistic force to drive cell motility

Although confined cells within a restricted space undergo morphological deformation owing to forces exerted by their surrounding environment, however, these deforming forces can also have an impact on cell motility. Such mechanical forces can be classified into two main models: the sling-shot model and the nuclear piston motion model.^79,80^ The sling-shot and nuclear piston models are two distinct theoretical concepts used to explain different aspects of cell migration, particularly in confined environments. While both models involve the mechanical aspects of cell movement, they focus on different mechanisms and phenomena [Figs. 2(c) and 2(d)].

The sling-shot model focuses on the storage and release of mechanical energy within a cell during migration through confined spaces.^79,81^ When a cell encounters a narrow passage, it undergoes deformation and polarization, storing mechanical energy, particularly in its actin filaments. Once the cell passes through confinement, it rapidly releases stored energy, resulting in a sudden recoil of the cytoskeleton. This release propels the cell forward with an increased velocity, facilitating migration after passing through the constriction. The model sheds light on how cells employ mechanical energy storage and release, showcasing an elastic recoil effect, to enhance migration through confined environments.^81^

In contrast, the nuclear piston model highlights the pivotal role of the cell nucleus as a piston-like structure that drives the cell forward during migration in confined spaces.^80,82^ When a cell encounters a narrow passage, it polarizes and attempts to fit its entire body through the confined area. However, the relatively large and rigid nucleus is an obstacle. To overcome this constraint, the cell generates mechanical forces that drive protrusion expansion to open a migration path in the micro-confinement environment. The cell nucleus acts like a piston in a syringe, pushing to pressurize the cell leading edge. This pressure initiates the formation of protrusions that serve as the initial point for the cell to navigate through the dense matrix. This piston-like movement of the nucleus propels the cell through narrow spaces, emphasizing its active participation in cell migration under confinement. The pressure generated during this process triggers mechanosensitive receptors within the cell membrane, propelling the cell to respond to the equilibrium of ion channel activity, hydraulic pressure, and osmotic pressure.^82^ When a cell is subjected to excessive pressure, it may experience deformation or rupture of the nucleus.^83^ Research suggests that increased contractility, combined with the nucleus being preferentially positioned toward the rear of the cell, leads to higher cytoplasmic pressure in the posterior region of the cell. This elevated pressure is believed to facilitate the passive movement of the nucleus through nuclear pore channels. The heightened nuclear influx subsequently subjects the nucleus to pressure, resulting in expansion, deformation of the nuclear envelope (NE), and eventual rupture. The movement characteristics of the cell nucleus vary depending on the geometric characteristics of the confined space.^84^

In summary, although both models consider the mechanical aspects of cell migration, they focus on different mechanisms. The sling-shot model emphasizes the storage and release of mechanical energy, which contribute to the rapid propulsion of the cell after passing through confinement. However, the nucleus piston model highlights the role of the nucleus as a piston-like structure that assists in pushing the cell forward during migration through confined spaces. Both models contribute to the understanding of cell migration in confined environments and demonstrate the complexity of the underlying mechanical processes.

The common features of cells during migration in a confined environment are mechanical stimuli such as tensile or shear stress and compression. These mechanical stimuli can activate the mechanical signaling pathways, which regulates cell migration patterns and cellular responses in the surrounding environment.

## V. IMPLICATION OF PHYSICAL STRESS ON INTRANUCLEAR MECHANICS

**FIG. 3.**
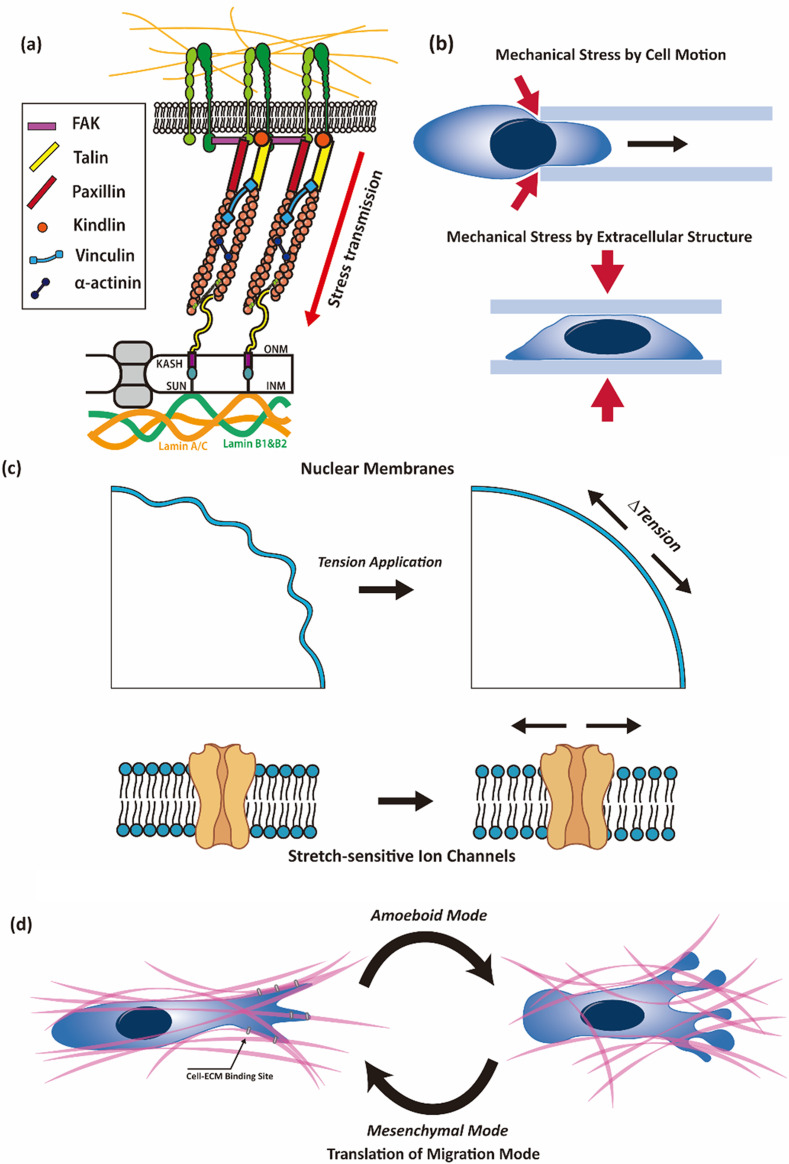
Cell responses and effects induced by mechanical stimulation. (a) The acceptance of mechanical stimuli from the external environment by cells through the LINC complex, connecting the cytoskeleton and the cell nucleus to the cell body, induces various internal changes within the cell. This mechanical stimulation leads to the deformation of the cell nucleus, instigating diverse alterations in the intracellular signals. (b) Mechanical stimulation in cells induces deformation through two primary mechanisms. First, physical stress is generated within the cell as it navigates narrow passages during active movement. Second, the cell undergoes direct external forces during geometric alterations, leading to deformation and subsequent compressive stress. (c) The cell detects nuclear deformation through an inherent sensing mechanism within the nucleus itself. Crucially, the key aspect of nuclear sensing lies in the alteration of nuclear tension during the transformation of the cell nucleus due to applied physical stress. This change in nuclear tension influences the activity of ion channels within the nuclear membrane. Consequently, this alteration plays a role in modifying cell mobility, thereby avoiding stress caused by cell deformation. (d) Environmental conditions, including physical factors in the surroundings of cells, significantly impact cell movement. One such factor is alterations in the size of the focal adhesion of the cell in response to changes in hydraulic pressure around the cell. Consequently, this modification influences the cell's migration mode, leading to transitions between mesenchymal and amoeboid modes based on pressure conditions.

Cells constantly interact with the external environment through various channels such as chemical and physical interactions. Notably, recent attention has been directed toward investigating cellular responses to mechanical stimuli originating from the external surroundings. Mechanotransduction is the ability of a cell to detect and respond to mechanical forces from its surroundings and convert them into biochemical signals. It is mediated, in part, by the linker of nucleoskeleton and cytoskeleton (LINC) complexes, which transmits mechanical signals from the cytoskeleton to the nucleus [Fig. 3(a)].^85^ The process of mechanotransduction involves several steps: mechanosensing, force transmission, signal transduction, and cellular response. This process affects cellular memory as cells retain information from previous mechanical experiences, thereby influencing their future behaviors and responses.

The LINC complex is a critical molecular bridge that connects the nuclear envelope (NE) to the cytoskeleton. It comprises two groups of proteins: SUN proteins in the inner nuclear membrane and KASH proteins in the outer nuclear membrane. The interaction between SUN and KASH proteins creates a physical link between the nucleoskeleton and cytoskeleton. This connection allows the transmission of mechanical forces from the cytoskeleton to the nucleus, and vice versa. The LINC complex plays a vital role in maintaining nuclear positioning, facilitating nuclear migration during cell division and migration, sensing mechanical cues from the environment, and regulating gene expression.

### A. The impact of cellular nuclear deformation on intracellular systems

The general case in which cells experience mechanical stress is cell migration in a confined microenvironment, such as interstitial migration,^86,87^ transmigration,^88,89^ or cancer cell metastasis.^77,90,91^ During cell migration in these confined spaces, cells often experience significant nuclear deformation, which is the alteration of the nuclear shape due to mechanical forces exerted on the nucleus [Fig. 3(b)]. The previous section outlined the various factors that contribute to the forces responsible for cell deformation. In this section, we explore additional intracellular mechanisms associated with alterations in the cell nucleus. One hypothesis explaining nuclear deformation during confined cell migration suggests that anchoring dynein and its regulator, Lissencephaly-1 (Lis1), to the NE via the LINC complex generates a forward-pulling force within the microtubule-centrosome system.^92^ This process relies on the LINC complex, linking the nucleus to a microtubule-centrosome network and incorporating the dynein adaptor Lis1. The contention is that nuclear deformation may occur in response to the resistance posed by matrix fiber contraction during confined cell migration induced by such a force.^93–95^

Owing to the deformability of the NE, the nucleus can assume various shapes, including elongation, compression, bending, and twisting, as the cell navigates through tight spaces. These changes in nuclear shape are reversible or irreversible depending on the magnitude and duration of the mechanical forces experienced by the nucleus. Nuclear recovery refers to the ability of a nucleus to return to its original shape after the removal or alteration of mechanical stress.^37,96^ Cells possess mechanisms for recovering their nuclear shape and integrity once they move out of a confined environment or experience reduced mechanical constraints. The LINC complex, along with other NE proteins, is responsible for restoring nuclear integrity during recovery. Mechanical cues that lead to nuclear deformation can also trigger various signaling pathways within the cell, leading to the activation of specific cytoskeletal and nuclear proteins involved in the repair process.

During cell migration in confined spaces, NE rupture and subsequent NE opening are common.^7,10^ If not addressed promptly, these abnormalities can escalate from DNA damage to outright cell death. This disturbance is expected to be particularly pronounced in regions where lamin A/C is compromised owing to heightened membrane curvature, intensifying its impact when lamin levels are reduced.^7,10^ To prevent cell death resulting from NE rupture, the cell activates an internal mechanism, swiftly repairing the damage through the facilitation of endosomal sorting complexes required for transport III (ESCRT-III).^7,10^ Experimental evidence confirms the rapidity of this repair phenomenon, which is typically completed within a 2-min timeframe.

The mechanism preventing NE rupture was also found in the retinoblastoma (Rb) and transformation-related protein 53 (p53) pathway, known as the tumor-suppressor pathway.^97^ This function is consistent with the situation in cancer cells in which metastasis must actively occur. This study showed that the lack of Rb or p53 could lead to NE rupture in HeLa, human bone osteosarcoma epithelial cells (U2OS), and human osteosarcoma cells (SJSA-1). However, following nuclear recovery, not all cells revert to their initial state. While some cells restore to their original state, they undergo substantial alterations within the nucleus during recovery process. Notably, deformation of the cell nucleus can induce DNA damage,^7,10,98,99^ irrespective of whether the NE is ruptured or intact. Deformation-induced DNA damage occurs during the S/G2 phase of the cell cycle and is specifically situated at replication forks.^100,101^ This finding suggests that mechanical forces can induce DNA damage and genomic instability.^101,102^

Research aimed at unraveling cellular responses to transformation suggests that the cell nucleus functions as a sensing apparatus [Fig. 3(c)].^103,104^ When cell compression surpasses the nuclear dimensions, it instigates outward expansion of the NE. The contraction is initiated when the NE attains a fully unfolded state. This alteration in the mechanical state of the NE and its membrane facilitates the release of calcium from the inner membrane, activating the calcium-dependent cytosolic phospholipase A2 (cPLA2). Recognized as a molecular sensor of nuclear membrane tension, cPLA2 plays a crucial role in the regulation of signal transmission and metabolism.^105,106^ Once activated, cPLA2 catalyzes the production of arachidonic acid,^107^ which enhances the adenosine triphosphatase activity of myosin II.^103,104^ This cascade induces the contraction of the actomyosin cortex,^108^ generating the force necessary for swift cell compression against physical pressure, particularly in a compressed microenvironment. This mechanism enables the cells to rapidly navigate and evade compressive forces.

### B. The influence of physical factors on cellular alterations

Cells exhibit adaptability to dynamic responses to changes of external environment. Notably, alterations in the extracellular fluid, which directly interacts with cells, can significantly affect the pattern of cell migration or cancer metastasis.^109^ An increase in viscosity due to increased mechanical loading results in the actin-related protein 2/3 (ARP2/3) complex-dependent dense actin network.^109,110^ This response triggers polarization of NHE1 through its interaction with ezrin, an actin-binding partner.^111,112^ NHE1 promotes cell swelling and increases membrane tension,^77^ activating TRPV4.^109^ This activation mediates calcium influx, and subsequently increases Ras homolog family member A (RHOA)-dependent cell contractility.^109,113^ Consequently, cells exhibit enhanced motility in environments with elevated viscosity.^109,114^

Furthermore, exposure to high viscosity endows cells with mechanical memory, resulting in altered dynamics in both the short and long term. This intricate interplay underscores the adaptability of cells to their surroundings and sheds light on the complex mechanisms governing the cellular responses to fluidic changes. Specifically, during cell migration in a confined environment, the phenotype can be influenced by hydraulic pressure [Fig. 3(d)].^65^ This process is regulated by TRPM7 and is recognized as a key mechanosensor of hydraulic resistance. The size of Arp2/3-dependent focal adhesions and the thickness of actomyosin were also controlled. Consequently, it has been observed that cells transition to either the amoeboid or the mesenchymal mode.

## VI. CONCLUDING REMARKS AND OUTLOOK

In this review, we comprehensively discussed cell movement in confined space, external physical factors influencing cell dynamics, and internal signaling pathways. We delved into studies that investigated how cells undergo transformation in response to various factors to shed light on their responses to physical stimuli. Mechanobiology, offering a perspective distinct from traditional biology, has prompted numerous endeavors to understand and harness cell movements in response to physical stimuli, particularly in controlled environments. Despite these efforts, numerous aspects of the physical properties of cells and their corresponding mechanisms of action remain unknown. In mechanobiology, which focuses on studying how cells respond to mechanical forces, particularly in controlled environments, there is a growing need for in-depth research to advance our understanding. This challenge persists, especially when it comes to directly observing stimuli and cellular reactions in the nucleus, adding a layer of complexity to the investigation.

This paper focuses on examining the deformation that occurs in the mechanical stimulation process of cells through external pressure, which implies the actual phenomena such as cancer cell metastasis and immune cell migration. It is also explicit to emphasize the importance of biological changes that cells undergo when subjected to external mechanical stimulation.

Moreover, through these studies, there is an opportunity to gain insights into the dynamic processes that govern the maintenance and alteration of cell shape and movement. By exploring these intricacies, researchers may uncover mechanisms to overcome challenges related to cell dynamics. This holistic approach contributes to a more comprehensive understanding of cellular behavior under mechanical stimuli, with potential implications for various fields, including cancer research and immunology.

Among the various components studied, the cell nucleus emerges as a pivotal player both biologically and mechanically. It undergoes diverse changes as cells interact with the external environment, necessitating appropriate controls. Understanding the precise characteristics of cells and addressing issues arising from deviations in their fundamental properties present numerous unresolved research topics. The intricacies of these topics warrant further exploration to unravel the mysteries surrounding the physical properties of cells and the complex mechanisms governing their responses.

Despite ongoing efforts by researchers to uncover these complexities, there are still many unknowns, and more strides are being made. As mentioned earlier, the development of mechanobiology is remarkably progressing, and this is expected to bring about significant changes in the future.

## Data Availability

The data that support the findings of this study are available within the article.
